# Progression-Free Survival Prediction in Small Cell Lung Cancer Based on Radiomics Analysis of Contrast-Enhanced CT

**DOI:** 10.3389/fmed.2022.833283

**Published:** 2022-02-24

**Authors:** Ningxin Chen, Ruikun Li, Mengmeng Jiang, Yixian Guo, Jiejun Chen, Dazhen Sun, Lisheng Wang, Xiuzhong Yao

**Affiliations:** ^1^Department of Radiology, The Second Affiliated Hospital of Fujian Medical University, Quanzhou, China; ^2^Department of Automation, Shanghai Jiao Tong University, Shanghai, China; ^3^Department of Radiology, Shanghai Institute of Medical Imaging, Zhongshan Hospital of Fudan University, Shanghai, China

**Keywords:** small cell lung cancer (SCLC), radiomics analysis, progression-free survival (PFS), contrast-enhanced CT, mediastinal window

## Abstract

**Purposes and Objectives:**

The aim of this study was to predict the progression-free survival (PFS) in patients with small cell lung cancer (SCLC) by radiomic signature from the contrast-enhanced computed tomography (CT).

**Methods:**

A total of 186 cases with pathological confirmed small cell lung cancer were retrospectively assembled. First, 1,218 radiomic features were automatically extracted from tumor region of interests (ROIs) on the lung window and mediastinal window, respectively. Then, the prognostic and robust features were selected by machine learning methods, such as (1) univariate analysis based on a Cox proportional hazard (CPH) model, (2) redundancy removing using the variance inflation factor (VIF), and (3) multivariate importance analysis based on random survival forests (RSF). Finally, PFS predictive models were established based on RSF, and their performances were evaluated using the concordance index (C-index) and the cumulative/dynamic area under the curve (C/D AUC).

**Results:**

In total, 11 radiomic features (6 for mediastinal window and 5 for lung window) were finally selected, and the predictive model constructed from them achieved a C-index of 0.7531 and a mean C/D AUC of 0.8487 on the independent test set, better than the predictions by single clinical features (C-index = 0.6026, mean C/D AUC = 0.6312), and single radiomic features computed in lung window (C-index = 0.6951, mean C/D AUC = 0.7836) or mediastinal window (C-index = 0.7192, mean C/D AUC = 0.7964).

**Conclusion:**

The radiomic features computed from tumor ROIs on both lung window and mediastinal window can predict the PFS for patients with SCLC by a high accuracy, which could be used as a useful tool to support the personalized clinical decision for the diagnosis and patient management of patients with SCLC.

## Introduction

Small cell lung cancer (SCLC) is an aggressive pulmonary neuroendocrine tumor that causes about 250,000 deaths worldwide yearly (1, 2). SCLC accounts for approximately 13% of lung cancers (3). SCLC is sensitive to both chemotherapy and radiotherapy, but has poor treatment performance with rapid recurrence, early metastatic dissemination, and poor prognosis (1, 4). Particularly, the overall survival (OS) rate and mortality of patients with SCLC have remained the same during the past several decades (5, 6). SCLC typically appears as a central tumor with hilar/mediastinal lymphadenopathy and distant metastases (7). The prognosis of SCLC strongly depends on the tumor stage. For example, the 5-year survival rate of limited stage is 20–25%, while only 2% for an extensive-stage (ES) disease (8).

In clinical practice, CT imaging is widely used for the clinical diagnosis of SCLC. However, CT-based SCLC diagnosis greatly depends on the experience and knowledge of radiologists. They can provide some qualitative analysis for SCLC prognosis by their experience, but the quantitative analysis of SCLC prognosis is generally lacked (9). In the clinical treatment of SCLC, etoposide–cisplatin or irinotecan–platinum remains the first choice for the first-line treatment of SCLC. Clinicians have observed that some patients made poor response to the chemotherapy (10, 11). It is clinically important to predict which patients with SCLC will have poor response to the chemotherapy, but this prognosis problem is seldom studied. In this paper, we try to construct a prediction model to quantitatively predict outcomes for patients with SCLC by their CT images scanned before treatment.

Recently, radiomics analysis is increasingly popular in tumor field, which is used for the diagnosis, stage, histology, prognosis, and treatment response assessment of various tumors (12, 13). By extracting a large number of image features from tumor regions, which depict the spatial heterogeneity in tumors, radiomics analysis is able to quantitatively characterize the tumor genomics and phenotypes, and identify tumor attributes that may be relevant to the tumor prognosis (14–16). However, due to the relatively low incidence of SCLC in all lung cancer subtypes, there were few reports focusing on the prognostic ability of radiomic features in SCLC. In regular diagnostic radiology, contrast-enhanced CT images are usually scanned for SCLC diagnosis. Radiologists observed CT images on both the lung window and mediastinal window. Here, an enhanced mediastinal window can provide more information for lung cancer diagnosis by providing clear tumor margin and enhancing pattern and intensity. It has been reported that the contrast-enhanced lung CT scan could influence the accuracy of pulmonary nodule classification (17, 18), and could independently predict the pathologic grade of lung adenocarcinoma (19). Additionally, researchers have mentioned the advantages of radiomics analysis in the prediction of response to chemotherapy in patients with SCLC (20), but radiomics analysis is performed only on the lung window of CT images. In such case, important radiomic features from the mediastinal window might be lost. This paper will study the progression-free survival (PFS) analysis in patients with SCLC by radiomics analysis on both the lung window and contrast-enhanced mediastinal window of CT images. By screening risk factors for SCLC prognosis and constructing a high-accuracy prediction model for outcomes of patients with SCLC, we will provide a useful tool for quantitatively predicting the PFS in patients with SCLC.

## Materials and Methods

### Patients

We retrospectively search the database from November 2012 to May 2019 under an active institutional review board, and written informed consents were acquired. The inclusion criteria were as follows: (1) patients pathologically proved SCLC by biopsy or operation. (2) Patients underwent chest CT scans. (3) Patients received standard Etoposide–Cisplatin combination chemotherapy. The exclusion criteria were as follows: (1) patients with treatment history before baseline CT scans; (2) patients lack of stage before treatment; (3) patients losing contact or die before progression; (4) patients without progression until deadline; (5) patients without contrast enhanced CT imaging; and (6) insufficient CT imaging. Finally, 186 cases were included in this study and divided into a training set (130 cases) and an independent test set (56 cases). The clinical information, such as age, gender, and stage were recorded.

### CT Imaging Parameters

All patients underwent contrast-enhanced chest CT on 64-slice multidetector row CT scanner (LightSpeed 64; GE Medical Systems, Milwaukee, WI, USA) with the following acquisition and reconstruction parameters: tube voltage of 120 kV; tube current of intelligent mAs; section thickness of 5 mm, and reconstruction interval of 5 mm. The contrast enhanced CT was administered intravenously with an amount of 60–70 ml of iohexol (Omnipaque 300; Amersham, Shanghai, China) followed by a saline flush of 20 ml, by using a power injector (LF CT 9000; Liebel-Flarsheim, Cincinnati, OH, USA) at a flow rate of 2.5–3.0 ml/s.

### Tumor Segmentation

A radiologist with 10 years of experience segmented each tumor region manually slice-by-slice on the axial CT images using the ITK-SNAP software (http://www.itksnap.org/pmwiki/pmwiki.php). Each tumor was segmented two times, first on the mediastinal window for consistency and then on the lung window, respectively. The lung window level (1600 and −500 HU) and the mediastinal window level (300 and 40 HU) were used during the tumor segmentation. The vessels and air regions were carefully excluded from the segmented tumor regions.

### Radiomic Feature Extraction

**Figure 1:**
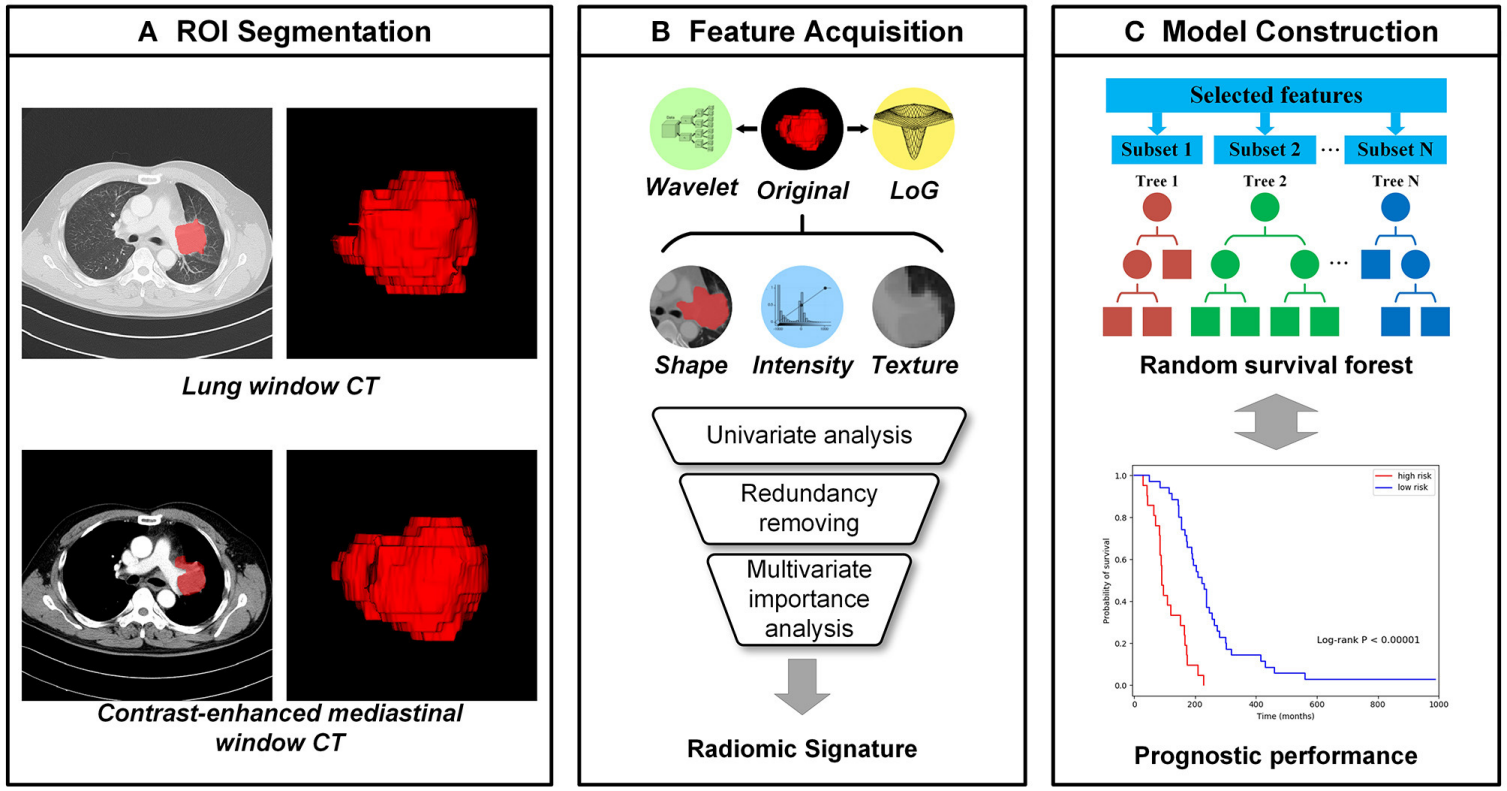
The workflow of radiomics analysis. **(A)** The tumor region of interests (ROIs), which were manually segmented by an experienced radiologist from CT images with the lung window and contrast-enhanced mediastinal window, respectively; **(B)** the high throughput image features were extracted automatically from each ROI and the radiomic signatures were selected from them; **(C)** Random survival forests (RSFs) models were established for the progression-free survival (PFS) prediction.

As shown in Figure 1, high through image features were automatically calculated from each tumor region using PyRadiomics package (21), including features describing shape, intensity, texture, etc. (22) Shape features reflect geometric properties of tumor regions. Intensity features were calculated using first-order statistics to depict the distribution of voxel intensities. Texture features could quantify the tumor heterogeneity and were calculated based on different texture matrixes. Additionally, wavelet filters and Laplacian of Gaussian (LoG) filters are applied to the original images for richer feature extraction (23). Wavelet features were extracted based on wavelet decomposition, which performed the multi-scale analysis of intensity and texture information. The LoG filters could enhance edge information, and 5 different sigma values were used to emphasize tumor textures of different coarseness. In total, 1,218 image features were extracted from each tumor region on the lung window and mediastinal window independently, and a total of 2,436 comprehensive CT image features were extracted.

### Feature Selection

Feature selection was conducted in the training set in three steps. First, the univariate prognostic ability of each feature was evaluated using a Cox proportional hazard (CPH) model (24). Features with concordance index (C-index) less than 0.5 were removed, because their prognostic abilities are worse than a random model:


(1) $$\begin{array}{l} F_{uni}=\left\{f|c\left(CPH\left(f,y\right)\right)>0.5,f\epsilon F\right\} \end{array}$$

Where, ***F*** is the unselected feature set, *c*(•) is the C-index of the CPH model which can be calculated by **Equation 4**. Second, to remove redundancy from image features, the variance inflation factor (VIF) was applied to quantify the collinearity between features (25). This procedure was performed iteratively until VIF values of all remaining features were less than the certain threshold, and the feature with the highest VIF value was removed in each iteration. The iteration process can be defined as:


(2) $$\begin{array}{l} F_{uni}=F_{uni}-\left(f|VIF\left(f,F_{uni}-f\right)>T_{vif}\right) \end{array}$$

Third, we used random survival forests (RSFs) for multivariate analysis to further simplify the features (26). An RSF model is an ensemble of tree-based learners which has powerful non-linear analysis capability. For each feature, an importance score was calculated based on the evaluation of relevance and prognosis of all features by RSF. Features with important scores above a certain threshold were finally selected and used to generate the radiomic signature:


(3) $$\begin{array}{l} F_{mul}=\left\{f|Score\left(RSF\left(f,F_{uni},y\right)\right)>T_{rsf},f\epsilon F_{uni}\right\} \end{array}$$

In above procedures, statsmodels (27), scikit-learn (28), and scikit-survival (29–31) packages were used for the implementation and grid search was performed to determine the parameters and thresholds. To compare the prognosis of different CT windows, the feature selection was performed on lung window features and mediastinal window features, respectively.

### Prognostic Model Establishment

Based on the selected radiomic features and three clinical features (gender, age, and stage), the random survival forests can be used to establish their corresponding prognostic model. For clinical features, selected lung window features and mediastinal window features, three different prognostic models can be generated, respectively. By combining the two classes of selected radiomic features into a feature set and simplifying them according to the Pearson's correlation (32), the corresponding prognostic models can be generated. By combining selected radiomic features and clinical features, the prognostic model can be constructed. For all above models, the 3-fold cross-validation was performed to determine model parameters in the training set.

### Statistical Analysis

A univariate analysis was used to assess the statistical significance of the clinical characteristics of the patients, and the independent sample *t*-test and χ^2^ test were performed for continuous and categorical variables, respectively. The correlation matrix of selected features was calculated based on Pearson's correlation coefficient and illustrated in a heatmap. The predictive capacity of radiomic signature was evaluated using Kaplan–Meier analysis (33). The performance of each prognostic model was evaluated on the independent test set with the C-index which is most frequently used in the survival analysis and assesses the overall prognostic ability of the model and can be defined as:


(4) $$\begin{array}{l} c=\frac{1}{num}\underset{i\;:\;\delta_{i}=1}{\sum }\underset{j\;:\;y_{i}<y_{j}}{\sum }I\left[r_{i}>r_{j}\right] \end{array}$$

Where, *i, jϵ*{1, ⋯ , *N*}, *num* denotes the number of all comparable pairs, δ_*i*_ is the binary event indicator, *I*[•] is the indicator function, and *r* is the risk predicted by the model. In addition, the time-dependent cumulative/dynamic AUC was calculated to measure the performance in a specific time range, which is an extension of the area under the receiver operating characteristic (ROC) curve (AUC) in survival data (34, 35). The cumulative/dynamic AUC (C/D AUC) at time *t* can be defined as:


(5) $$\begin{array}{l} A\hat{U}C\left(t\right)=\frac{\sum_{i=1}^{n}\sum_{j=1}^{n}I\;[y_{j}>t]\;I\;[y_{i}<t]\;\omega_{i}I\;[r_{i}>r_{j}]\;}{\left(\sum_{i=1}^{n}I\;[y_{i}>t]\right)\left(\sum_{i=1}^{n}I\;[y_{i}\leq t]\;\omega_{i}\right)} \end{array}$$

Where, ω_*i*_ is inverse probability of censoring weights (IPCW). All statistical analyses were two-sided with the statistical significance level of 0.05, and performed with statsmodels, scikit-learn, and scikit-survival packages in Python 3.6.

## Results

### Clinical Characteristics

A total of 186 cases were enrolled and divided into a training set and an independent test set. As shown in Table 1, the median (range) of age in the two sets were 62 (37–80) and 62 (43–78). In training set, 109 (83.8%) cases were men and 21 (16.2%) cases were women, with 47 (36.2%) cases were limited stage and 83 (63.8%) cases were extensive stage. In the test set, there were 50 (89.3%) male cases and 6 (10.7%) female cases, and 19 (33.9%) and 37 (66.1%) cases were limited and extensive stage, respectively. There were no significant differences for all clinical characteristics between the training and test sets (*p* = 0.334–0.771). In addition, through the univariate survival analysis, stage (*p* = 0.007) and sex (*p* = 0.039) were statistically relevant to the survival, and there was no significant difference regarding to age (*p* = 0.698).

**Table 1:** Clinical characteristics.

**Characteristics**	**Training set**	**Test set**	***p*-value**
Number	130	56	
Gender			
Male	109 (83.8%)	50 (89.3%)	0.334
Female	21 (16.2%)	6 (10.7%)	
Age	62 (37–80)	62 (43–78)	0.635
Stage			
Limited stage	47 (36.2%)	19 (33.9%)	0.771
Extensive stage	83 (63.8%)	37 (66.1%)	

During the follow-up, different types of progression were observed. Among 186 cases, there were 85 (45.7%) cases of primary progression, 23 (12.4%) cases of original metastases progression, 13 (7.0%) cases of primary progression and newly metastases, 11 (5.9%) cases of both the primary and metastasis progression, 16 (8.6%) cases of newly brain metastases, 14 (7.5%) cases of newly bone metastases, 8 (4.3%) cases of newly lung metastases, 6 (3.2%) cases of newly liver metastases, 1 (0.5%) case of cardiac metastases, 1 (0.5%) cases of pancreas metastases, 3 (1.6%) cases of adrenal metastases, and 5 (2.7%) cases of newly multiple metastases.

### Important Radiomic Feature Selection

**Figure 2:**
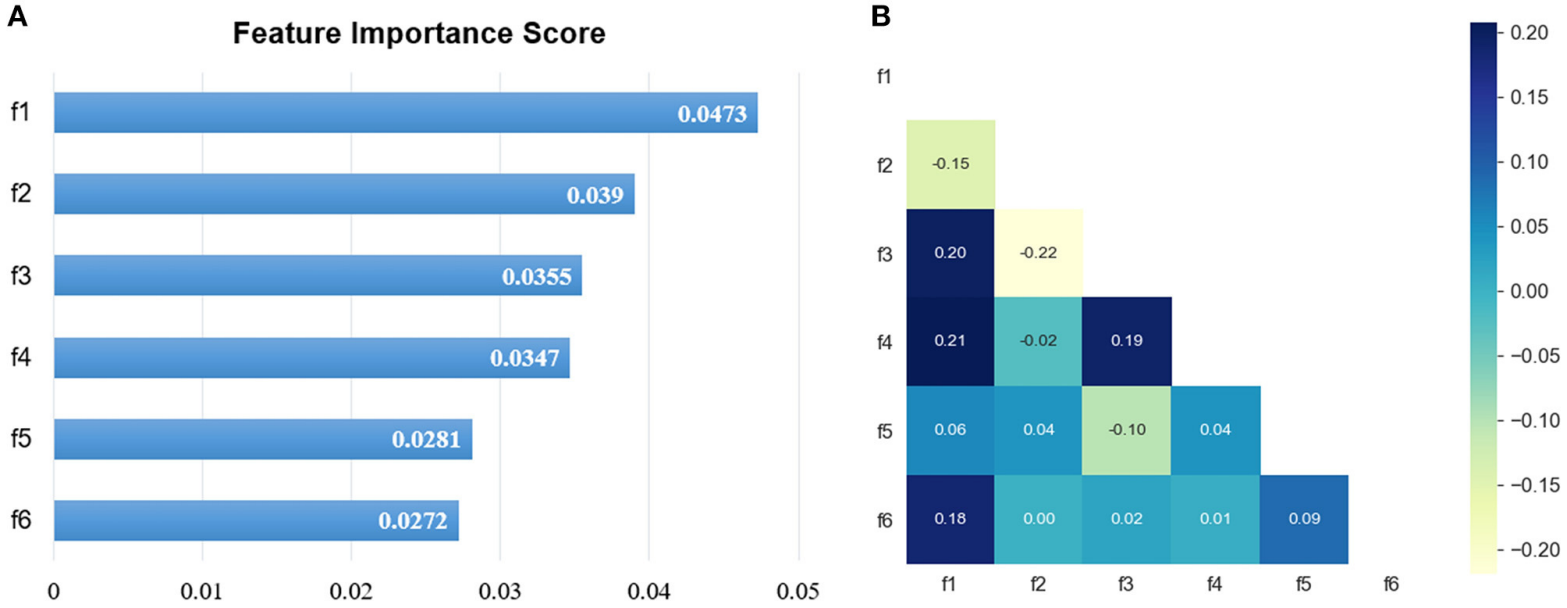
The selected lung window features. **(A)** The RSF-based feature importance score; **(B)** the correlation heatmap.

**Figure 3:**
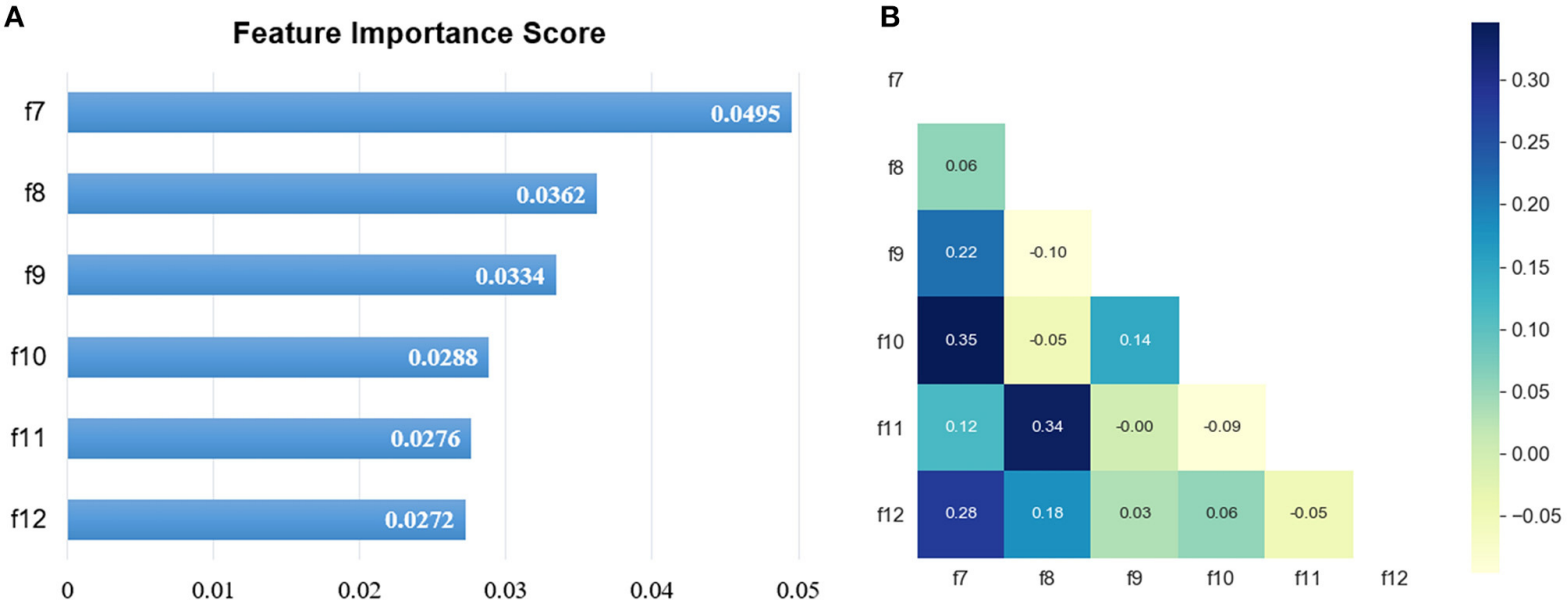
The selected mediastinal window features. **(A)** The RSF-based feature importance score; **(B)** the correlation heatmap.

In total, 1,218 radiomic features were extracted from CT images on the lung window and mediastinal window, respectively, and the feature selection was performed independently on both windows. After the univariate analysis, 724 and 895 features were remained, respectively. Then, with an iterative collinearity elimination based on VIF, a mass of redundant features was removed, resulting in 17 and 16 remained features for the lung window and mediastinal window, respectively. Finally, an RSF-based multivariate analysis was performed on the remaining features, and 6 features for the lung window (f1, f2, f3, f4, f5, and f6) and 6 features for the mediastinal window (f7, f8, f9, f10, f11, and f12) were selected as radiomic signature, as shown in Table 2 and Figures 2, 3. The correlation heatmaps in Figures 2, 3 indicated that the selected features are relatively independent. Definitions of all selected features are in compliance with the Imaging Biomarker Standardization Initiative (IBSI) (36).

**Table 2:** Description of the selected radiomic features.

**Index**	**Feature**	**Description**
F1	Lung_log-sigma-1-0-mm-3D_glcm_Correlation	A Measure of the linear dependency of gray level values to their respective voxels in the GLCM
F2	Lung_log-sigma-3-0-mm-3D_glcm_ClusterShade	A measure of the skewness and uniformity of the GLCM
F3	Lung_original_firstorder_90Percentile	The 90th percentile of the voxels included in the ROI
F4	Lung_wavelet-LHH_glcm_ClusterShade	A measure of the skewness and uniformity of the GLCM
F5	Lung_log-sigma-4-0-mm-3D_glszm_SmallAreaLowGrayLevelEmphasis	A measure of the proportion of the joint distribution of smaller size zones with lower gray-level values
F6	Lung_original_shape_Flatness	A measure of the relationship between the largest and smallest principal components in the ROI shape
F7	Mediastinal_log-sigma-4-0-mm-3D_glszm_SmallAreaLowGrayLevelEmphasis	A measure of the proportion of the joint distribution of smaller size zones with lower gray-level values
F8	Mediastinal_wavelet-HHL_firstorder_Skewness	A measure of the asymmetry of the distribution of values about the Mean value
F9	Mediastinal_original_shape_Flatness	A measure of the relationship between the largest and smallest principal components in the ROI shape
F10	Mediastinal_log-sigma-5-0-mm-3D_glszm_GrayLevelNonUniformityNormalized	A measure of the variability of gray-level intensity values in the image
F11	Mediastinal_wavelet-HLH_firstorder_Skewness	A measure of the asymmetry of the distribution of values about the Mean value
F12	Mediastinal_log-sigma-3-0-mm-3D_glcm_InverseVariance	A measure of inverse variance

### Performance of Prognostic Model

**Figure 4:**
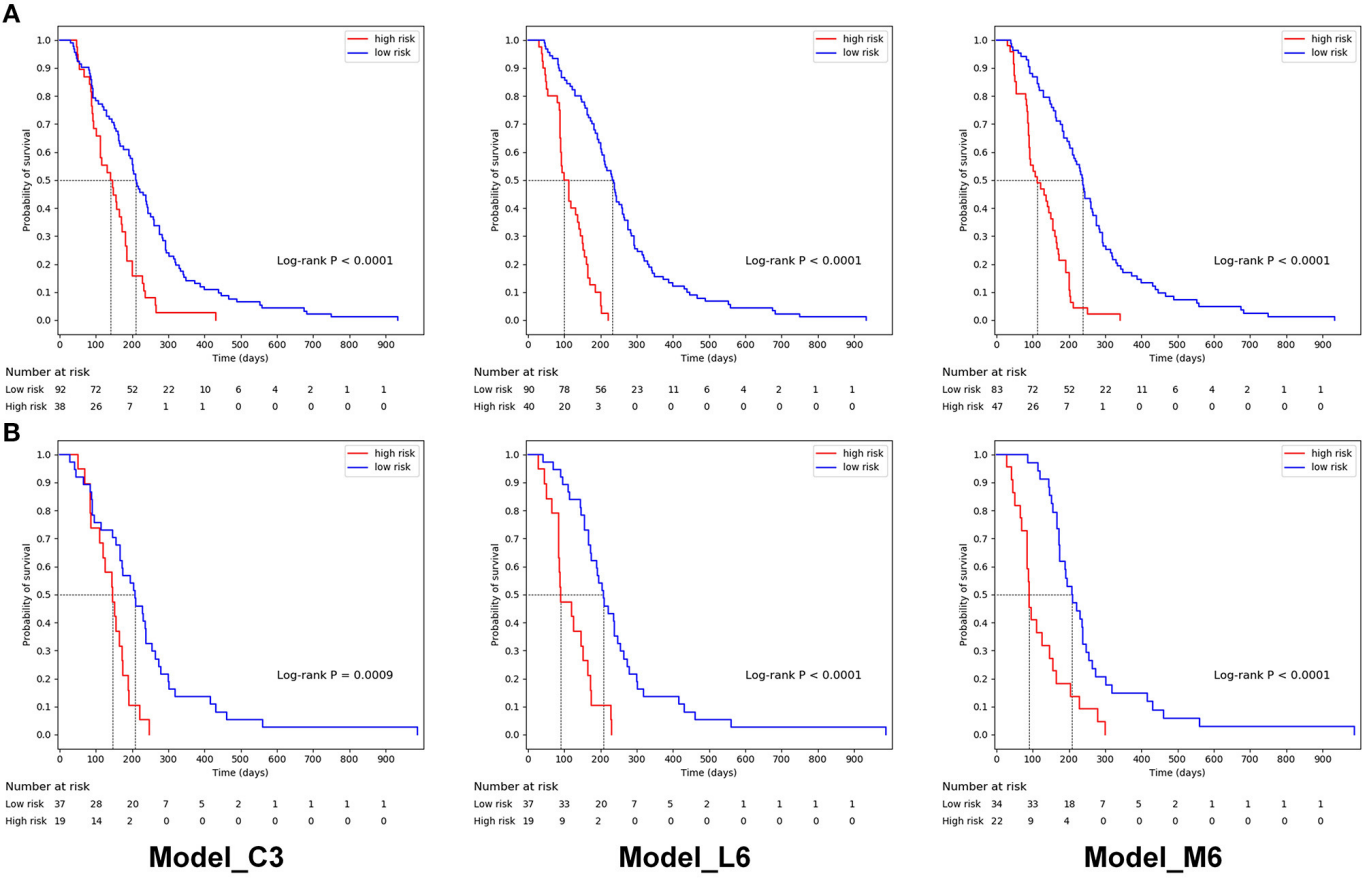
Kaplan–Meier progression-free survival curves of prognostic models based on 3 clinical features (Model_C3), 6 lung window features (Model_L6), and mediastinal window features (Model_M6) for patients in **(A)** the training set and **(B)** the independent test set. In the training set, the median PFS were 211, 233, and 238 days for the low-risk group and 140, 99, and 112 days for the high-risk group. In the independent test set, the median PFS were 209, 209, and 209 days for the low-risk group and 147, 91 and 90 days for the high-risk group.

Based on 3 clinical, 6 lung window, and 6 mediastinal window features, three basic prognostic models can be established, respectively, namely, Model_C3, Model_L6, and Model_M6. For these basic models, the optimal risk cut-off was determined using log-rank test on the training set, and patients in the training set and test set were stratified into high-risk and low-risk groups using the same cut-off, respectively. In Figure 4, the Kaplan–Meier curves of lung window (cut-off = 112) and mediastinal window (cut-off = 117) features revealed the significant difference in PFS between high-risk and low-risk groups, which is better than clinical features (cut-off = 112), demonstrating the predictive capabilities of the two different radiomic signatures.

By combining the lung window and mediastinal window radiomic features into a feature set, the combined model based on all 12 radiomic features (Model_L6+M6) was established. Additionally, to simplify radiomic features and reduce the risk of overfitting, we removed features with the higher correlation between two feature sets (f6, which has a Pearson's correlation coefficient of 0.75 with f9 and a lower importance score). Then, 11 remained radiomic features were used to generate a simplified model (Model_L5+M6). The overall prognostic ability of each prognostic model was evaluated with C-index and illustrated in Table 3. The C-index values of Model_C3, Model_L6, Model_M6, Model_L6+M6, and Model_L5+M6 are 0.6426, 0.7455, 0.7728, 0.7927, and 0.8033 for the training set and 0.6026, 0.6951, 0.7192, 0.7362, and 0.7531 for the test set, respectively. Model_L5+M6 attains the highest C-index among the models. In addition, by combing the selected radiomic features and clinical features, their corresponding prognostic models have better performances or the performances comparable with the radiomic features-based models.

**Table 3:** Prognostic performance of different survival prediction models.

**Model**	**C-index**		**C/D AUC_90**	**Mean C/D AUC**
	**Training set**	**Test set**		
Basic models				
Model_C3	0.6426	0.6026	0.5218	0.6312
Model_L6	0.7455	0.6951	0.7727	0.7836
Model_M6	0.7728	0.7192	0.8646	0.7964
Combined models based on radiomic features				
Model_L6+M6	0.7927	0.7362	0.8769	0.8387
**Model_L5+M6**	0.8033	**0.7531**	**0.8902**	**0.8487**
Combined models based on radiomic and clinical features				
Model_L6+C3	0.7500	0.7316	0.7898	0.8206
Model_M6+C3	0.7933	0.7440	0.8485	0.8367
Model_L6+M6+C3	0.7961	0.7459	0.8523	0.8413
Model_L5+M6+C3	**0.8276**	0.7518	0.8258	0.8441

*The bold values indicate the highest score in each performance metric. C3 means 3 clinical features, L6 means 6 selected lung window radiomic features, M6 means 6 selected mediastinal window radiomic features, L5 means the remained 5 lung window radiomic feature by removing the most correlated feature (f6) among the 12 radiomic features according to Pearson's correlation on the basis of L6*.

The C/D AUC can evaluate the model performance from time level on the test set. The C/D AUC_90 evaluates how well these models can distinguish the patients progressing before and after 90 days, which is an important time for the prognosis of SCLC. As illustrated in Table 3, Model_L5+M6 achieved the best C/D AUC_90 of 0.8902. Furthermore, the restricted mean C/D AUC (37), which is a summary measure of discrimination ability of each model, was calculated. The mean C/D AUC of Model_C3, Model_L6, Model_M6, Model_L6+M6, and Model_L5+M6 are 0.6312, 0.7836, 0.7964, 0.8387, and 0.8487, respectively. These results are consistent with C-index results of these models.

**Figure 5:**
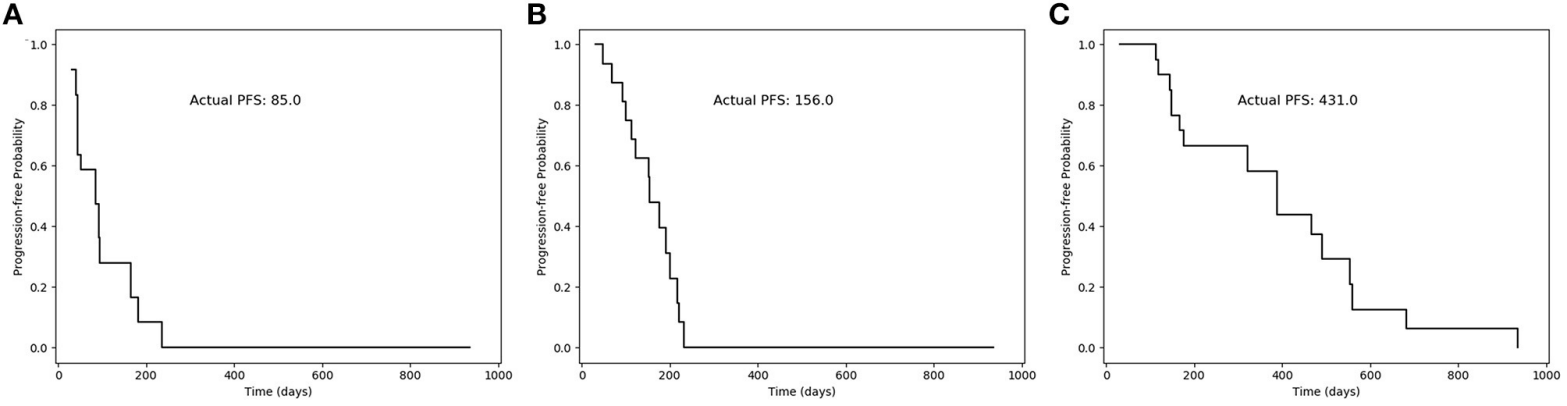
Progression-free probability curves of the survival function generated by our model for three typical cases. **(A)** A patient with PFS less than 90 days (85 days); **(B)** a patient with PFS more than 90 days (156 days); and **(C)** a patient with PFS more than 1 year (431 days).

Based on our survival models, a survival function can be generated and used to analyze the prognosis for each patient. Three typical cases with different survival time were illustrated in Figure 5. The survival function gives the progression-free probability at different times, which can provide clinicians with a more intuitive and reliable prognostic prediction and is helpful for the personalized clinical decision-making.

**Figure 6:**
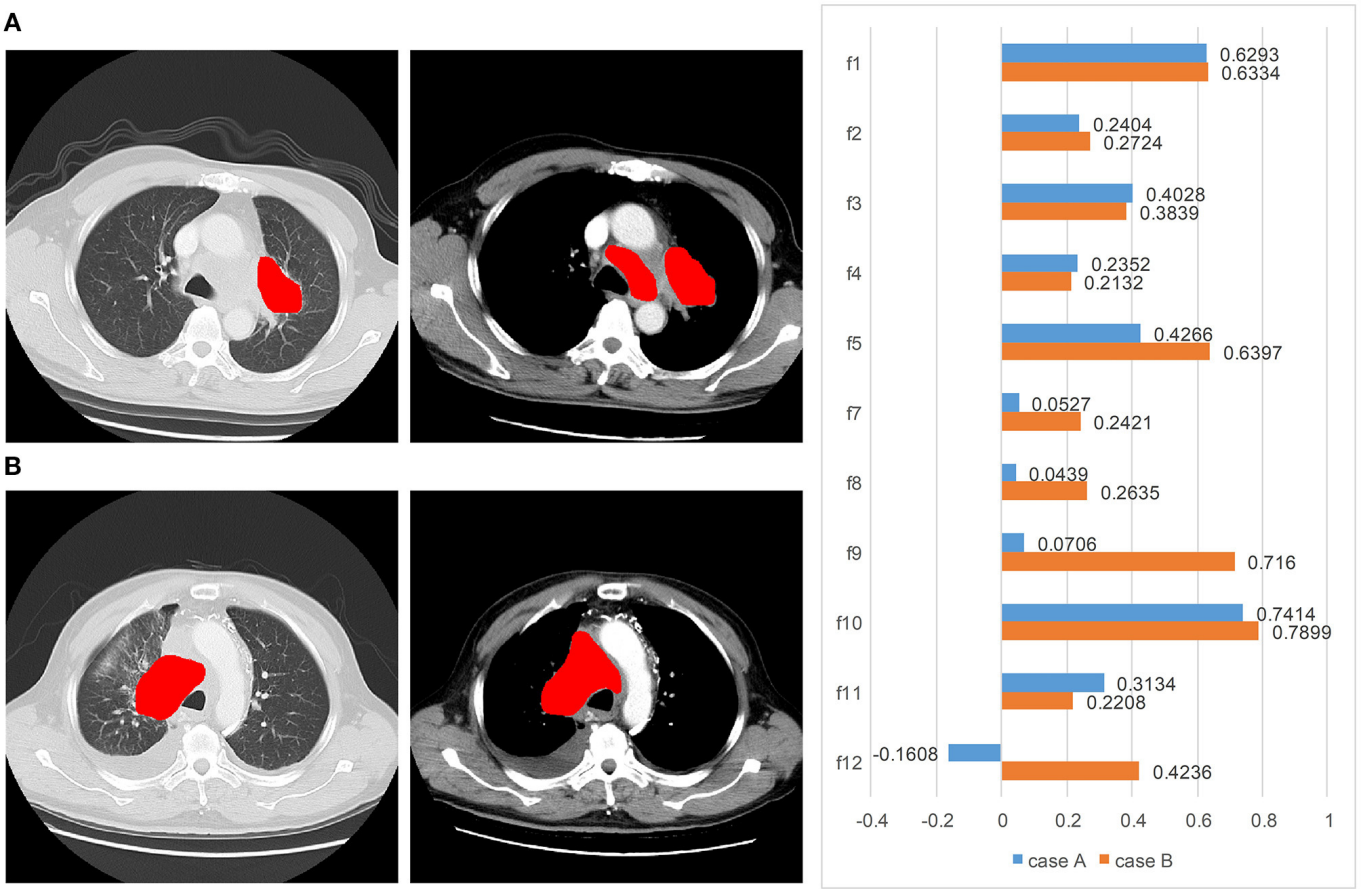
Two typical cases from the visual analysis and our method: **(A)** a patient (male, age = 62 years, limited stage) with PFS of 90 days. Our model correctly predicted it with a high risk of 157.0, but the visual and clinical prognosis was good. **(B)** A patient (male, age = 67 years, extensive stage) with PFS of 431 days. Our model correctly predicted it with a low risk of 55.0, but the visual and clinical prognosis was poor. From left to right are the lung window CT, enhanced mediastinal window CT, and the feature weights, respectively.

Two typical cases which are challenging and hard to predict the prognosis are illustrated in Figure 6. For case A, a pulmonary tumor with mediastinal and lateral hilar lymph node metastases was found on CT images. No distant metastasis was detected. This 62-year-old man was diagnosed with a good prognosis based on the visual analysis and limited stage. However, the actual PFS was only 90 days, and our model correctly predicted it with a high risk of 157.0. For case B, in a 67-year-old man, bone metastases and lymph node metastases were found on the primary radiological scans. The primary pulmonary tumor of case B, with obstructive pneumonia, was larger than the case A. This patient was diagnosed as a poor prognosis with extensive stage. However, the actual PFS was over a year (431 days), and our model correctly predicted it with a low risk of 55.0.

## Discussion

In this study, a PFS predictive model was proposed by integrating the radiomic features extracted from lung window and mediastinal window CT images in patients with SCLC. Our main findings indicated the contrast enhanced-mediastinal window radiomic features as an independent reliable prognostic factor. Our model effectively separated the groups of high risk and low risk, gave better predictive performance than the typical clinical visual analysis, and generate a survival function to analyze the prognosis for each patient. Our radiomics-based model offers more reliable PFS predictions which could support the personalized clinical decision-making.

Radiomics can extract more quantitative information than bare eye to guide clinical decisions, usually from non-enhanced CT images. Unenhanced CT images reflect the tumor heterogeneity and microenvironment, demonstrating the prognostication and treatment response. Fried et al. (38) and Ganeshan et al. (39) indicated that texture features from pretreatment non-contrast CT scans may provide the prognostic information for patients with non-small cell lung cancer (NSCLC). Mohammadhadi Khorram et al. (40) stated that radiomics was useful for predicting the early-stage NSCLC recurrence, progression, and recurrence free survival. For patients with SCLC, Haifeng Wei et al. (10) revealed radiomic texture characteristics may be an independent predictor of the efficacy of chemotherapy and help clinical guidance. Additional enhanced contrast mediastinal images may provide more image information about the grade of enhancement and the heterogeneity of the tumor, which may be due to the presence of different tumor vascularization (41). Radiomic texture analysis on the contrast-enhanced CT could be a good predictor of the survival and treatment response in patients with NSCLC (42, 43). Another study stated that the texture analysis of CECT images provides the predicted pathologic grading of lung adenocarcinoma (19).

In this study, we investigated the value of radiomic signature in predicting the prognostication and treatment response in patients with SCLC. Two cases with different prognosis are shown in Figure 6. The prognosis was contrary to the expectation with clinical visual analysis. Compared with the inadequate clinical outcomes, our radiomic model with the contrast–enhanced mediastinal window predicted an accurate outcome with little difference.

Our radiomic models showed a good performance of C-index in predicting the PFS with conventional lung window features and enhanced mediastinal window features respectively, with a lowest C-index of clinical model. Furthermore, the predictive performance of radiomic features in an enhanced mediastinal window was superior to the lung window, indicating that enhanced mediastinal window features played an important role in predicting the PFS in patients with SCLC. Some previous studies showed that the predictive performance of quantitative texture features were similar between non-contrast and contrast-enhanced CT in diagnosing lung nodule (17, 44). Another study reported that the unenhanced CT was better than the contrast-enhanced CT on the predictive performance. However, those studies focused on lung adenocarcinoma and did not include SCLC cases which may cause result deviation (45). Our study results were generally in agreement with some other studies. Linning et al. (18) and Liu et al. (19) investigated that the contrast-enhanced CT were useful predictors of survival and treatment response, which may be related to the tumor heterogeneity. After contrast administration, tumoral vascularity may reflect local spatial variations in image brightness, and then result in the variability of radiomic features (18, 46).

For further verification, we analyzed the time-dependent cumulative/dynamic ROC curve and calculated the time-dependent AUC. The time-dependent cumulative/dynamic ROC curve analysis defined a marker value updated at each time point during the disease status individually, allowed to compare the marker's predictive ability and may give guidance for medical decisions (47). In our study, we achieved similar results with the C-index, suggesting the importance of enhanced-mediastinal window in predicting the PFS. Our study illustrated an important time of 90 days for the prognosis of SCLC and the median survival time was around 200 days in high-risk groups from Kaplan–Meier curves.

There were numerous texture features in CT images, which may provide different anatomical and biological information in tumor. Thus, the selection of texture features was meaningful and time-saving for model building. During feature extraction, original images were transformed to derived images with the wavelet filter and LoG filter to extract more radiomic features. After feature selection, the most important features for lung window and mediastinal window were selected respectively. For both lung window and mediastinal window, 50% (3/6) selected features were LoG-based, including the top-ranked features. These results illustrated that LoG-based features play a more important role in the survival analysis of SCLC. Actually, a LoG filter could enhance the edge information by emphasizing the areas of gray level change. The fine textures and coarse textures of the tumor were both taken into consideration by high and low sigma parameter. During the clinical diagnosis of SCLC based on CT images, the texture feature of the tumor edge is always an important indicator, which also proves the rationality of extracted radiomic features.

There were several limitations in our study. First of all, due to the relatively low incidence of SCLC in Asian race, the population of this study is relatively small. Second, previous studies indicated that the suitable section thickness may be as thin as 1.25 or 2.5 mm. But the section thickness was 5 mm in this retrospective study, which may reduce the predictive accuracy of lung window. Third, smoking is an important risk factor in patients with SCLC especially in women, which did not calculate into the clinical features in our study.

## Conclusion

In summary, our study revealed that the textual features extracting from the contrast-enhanced mediastinal window were useful for predicting the PFS. The integration of textual features from the lung window and contrast-enhanced mediastinal window provided the more valuable information in survival prediction in comparison with the conventional visual assessment, which could be applied to support personalized clinical decision for the diagnosis and patient management in patients with SCLC.

## Data Availability Statement

The raw data supporting the conclusions of this article will be made available by the authors, without undue reservation.

## Ethics Statement

The studies involving human participants were reviewed and approved by Zhongshan Hospital of Fudan University. The patients/participants provided their written informed consent to participate in this study.

## Author Contributions

XY and LW designed and conducted the study. NC, RL, XY, and LW contributed to the manuscript writing process. NC and RL prepared the first draft of the entire manuscript. NC performed tumor segmentation. RL, DS, and LW contributed to the radiomic feature extraction, feature selection, and data analysis. NC, MJ, YG, and JC contributed to data interpretation. All authors provided the final approval of the version submitted for publication.

## Funding

NC reports funding from the Medical and Health Foundation for Young Scientists of Fujian Province (Grant No. 2020QNA059).

## Conflict of Interest

The authors declare that the research was conducted in the absence of any commercial or financial relationships that could be construed as a potential conflict of interest.

## Publisher's Note

All claims expressed in this article are solely those of the authors and do not necessarily represent those of their affiliated organizations, or those of the publisher, the editors and the reviewers. Any product that may be evaluated in this article, or claim that may be made by its manufacturer, is not guaranteed or endorsed by the publisher.
